# Deep Learning Approach for Species Identification of Forensically Important *Sarcophagid flies* (Diptera: Sarcophagidae) in China

**DOI:** 10.3390/insects17040374

**Published:** 2026-04-01

**Authors:** Sen Hou, Jiali Su, Xinyi Yao, Xinglin Li, Jinliang Du, Jianxia Li, Futeng Jiang, Yang Xia, Shuguang Zhang, Wen Cui, Yequan Wang, Lipin Ren

**Affiliations:** 1 School of Forensic Medicine, Jining Medical University, Jining 272067, China; 2 College of Computer Science, Nankai University, Tianjin 300350, China; 3 Department of Forensic Science, School of Basic Medical Sciences, Central South University, Changsha 410083, China; 4 Department of Forensic Science, School of Basic Medical Sciences, Inner Mongolia Medical University, Hohhot 010110, China; 5 Shanghai Key Lab of Forensic Medicine, Key Lab of Forensic Science of Ministry of Justice, Shanghai 570100, China

**Keywords:** forensic entomology, sarcophagid flies, image classification, deep learning, ViT-LoRA

## Abstract

Accurate species identification of necrophagous flies is fundamental to forensic entomology, particularly for postmortem interval (PMI) estimation in decomposed remains. Here, we conducted a targeted carrion-baited survey along the Shandong Peninsula and documented 15 *Sarcophaga* species, including the first regional records of *S. cinerea*, *S. pingi*, and *S. pterygota*. We established an expert-validated image dataset for automated identification. We then developed a parameter-efficient identification framework by fine-tuning a pretrained Vision Transformer with Low-Rank Adaptation (ViT-LoRA) on this custom dataset. Compared with conventional CNN-based models, ViT-LoRA achieved 98.50% species-level accuracy while updating only ~0.16 M trainable parameters, and it converged rapidly and stably within ~10 epochs, demonstrating efficient adaptation under limited training data. This study provides faunistic and distributional data on carrion-associated *Sarcophaga* species in the coastal Shandong Peninsula, characterizes their regional distribution patterns, and offers a scalable image-based identification approach for forensically important sarcophagid flies.

## Simple Summary

Accurate identification of flesh flies is essential in forensic entomology because different species develop at different rates, which can affect postmortem interval (PMI) estimation. In this study, we documented 15 *Sarcophaga* species from carrion-baited surveys along the coastal Shandong Peninsula, including three species newly recorded from this region. To support faster and more reliable identification, we established an expert-validated image dataset and developed a lightweight deep-learning framework based on Low-Rank Adaptation of a pretrained Vision Transformer (ViT-LoRA). ViT-LoRA achieved 98.5% species-level accuracy while updating only ~0.16 million trainable parameters and converged rapidly within ~10 epochs. Overall, this study provides new faunistic and distributional data on carrion-associated *Sarcophaga* species in the coastal Shandong Peninsula and demonstrates the potential of a scalable image-based approach for species identification in forensically important sarcophagid flies.

## 1. Introduction

The family Sarcophagidae, commonly known as flesh flies, comprises approximately 2300 described species worldwide [1,2]; the subfamily Sarcophaginae are regularly associated with decomposing remains and are of particular forensic importance, with *Sarcophaga* being one of the most species-rich genera [3,4]. Most sarcophagids are ovoviviparous, depositing first-instar larvae rather than eggs directly onto carrion [5,6]. Species in the subfamily Sarcophaginae are of particularly forensic significance because their predictable colonization and development patterns provide critical evidence for PMI estimation [2,7,8,9,10,11]. Accurate species-level identification represents a critical priority in forensic entomology. Traditional identification relies primarily on adult morphology and remains the gold standard for well-preserved specimens, but it requires specialized taxonomic expertise that hinders several critical challenges still remain its accessibility and efficiency. Meanwhile, the declining number of trained taxonomists further exacerbates these limitations. Additionally, its reliability declines markedly when specimens are damaged, incomplete, or when closely related and cryptic taxa are involved [12]. To overcome these challenges, molecular approaches, particularly DNA barcoding, have significantly improved the accuracy and precision of species identification, enabling reliable discrimination of closely related species, even from degraded or fragmented specimens [13]. Mitochondrial genomes (mitogenomes) have further refined phylogenetic resolution within Sarcophaginae [14,15,16,17,18]. However, these methods remain labor-intensive, time-consuming, and expensive. Furthermore, DNA extraction is inherently destructive, rendering the specimen unusable for subsequent analysis.

Recent advances in computer vision and deep learning offer a complementary, non-destructive route for rapid and broadly accessible species identification [19,20]. Convolutional neural networks (CNNs) have emerged as the predominant algorithm due to their ability to learn discriminative image features without manual engineering [21,22], enabling high-throughput identification from images [23]. Image-based deep learning has achieved strong performance across diverse insect groups. For example, Simović et al. (2024) developed a CNN model capable of automatically classifying 90 taxa of aquatic insects, achieving an impressive accuracy of 98.7% [24]. Similarly, Wang et al. (2021) integrated Mask R-CNN with discriminative deep metric learning (AlexNet) to identify 83 fruit fly species based on wing images, achieving the best classification accuracy up to 90% [25]. Additionally, Kittichai et al. (2021) employed YOLO-derived models to simultaneously localize and classify field-caught mosquito images by species and sex, achieving a mean average precision of 99% and sensitivity of 92.4% [26]. In forensic entomology, Apasrawirote et al. (2022) evaluated ResNet-101, DenseNet161, VGG19, and AlexNet for maggot identification from posterior spiracles and developed an associated web-based tool [19]. Gohe et al. (2024) applied MobileNetV3-Large and VGG19 for classifying adult flies from families Calliphoridae, Sarcophagidae, and Rhiniidae, achieving accuracies of 99.39% and 99.79%, respectively, highlighting the applicability of these models in forensic investigations [27]. Moreover, Ma et al. (2025) introduced the Global–Local Balanced Vision Transformer (GLB-ViT), integrating CNN’s local feature extraction capabilities with transformers’ global context understanding, achieving a remarkable overall accuracy of 94% in classifying ten sarcosaphagous flies [28].

In general, these studies highlight the potential of image-based identification systems to preserve specimen integrity and reduce subjectivity and inter-observer variability [29]. Moreover, they are potentially cost-effective and accessible, requiring only available imaging devices (e.g., smartphones or portable microscopes). Nonetheless, several critical challenges still remain, high-quality, expertly annotated image datasets for medically and forensically important taxa, especially at regional scales are still scarce [19,20]. In addition, most previous studies focus on morphologically distinct species across different families, in which interspecific morphological differences are relatively pronounced, potentially overestimating classification performance. In contrast, accurate identification among closely related species within the same family, where morphological similarity is high, remains a critical and underexplored challenge. Therefore, expanding publicly accessible, rigorously annotated image databases and integrating deep learning-based image recognition with molecular methods, such as DNA barcoding, represent promising strategies to improve diagnostic accuracy [25]. In this study, we aimed to survey carrion-associated *Sarcophaga* species along the Yellow Sea–Bohai Sea coast, establish an expert-validated image dataset comprising 15 species, and assess whether a parameter-efficient ViT-LoRA could improve fine-grained species identification under limited training data [30,31].

## 2. Materials and Methods

### 2.1. Resources of Adult Specimens and Image Dataset

From June to October 2024 and 2025, adult specimens of 15 *Sarcophaga* species were collected along the coastal Shandong Peninsula in China (Yellow Sea to the Bohai Sea), using decomposed pig lungs as bait (Table 1 and Figure S1). All specimens were euthanized by freezing and subsequently identified by an expert dipterological taxonomist (Lushi Chen) using traditional morphological keys under a dissecting microscope [32]. Voucher specimens were preserved in 75% ethanol and subsequently stored at −20 °C with unique identifiers deposited in Ren’s Lab. Except for *S. crassipalpis* and *S. polystylata*, for which only males were available due to physical damage and insufficient image quality, three males and three females were initially selected for the remaining species. In total, 82 adult individuals were retained for model development. All specimens were photographed as single-view images using a Nikon SMZ1270 microscope imaging system (Tokyo, Japan). A total of 2631 original high-resolution images were acquired to capture intra-specific morphological variability (Figure S2). To improve robustness to viewpoint and orientation variation, data augmentation was applied only to the training set, including random resized cropping, horizontal flipping (*p* = 0.5), and random rotation (±15°). After preprocessing and augmentation, 6373 images were used for model development. Importantly, to avoid information leakage across individuals, the dataset was split into training and test sets at an 8:2 ratio using an individual-level strategy, ensuring that images from the same specimen were never simultaneously assigned to both sets.

### 2.2. Model Training and Evaluation

**Figure 1:**
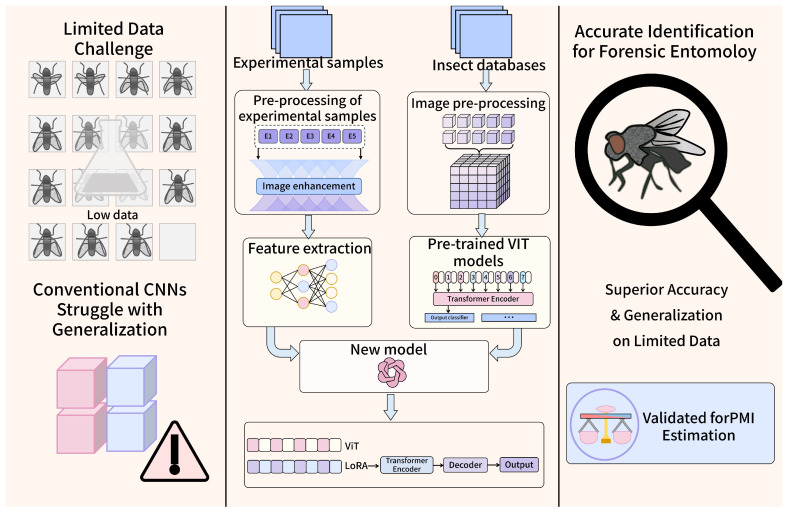
Schematic of the ViT-LoRA architecture for species identification of forensically important sarcophagid flies under limited data. The curated images from experimental and external insect databases are preprocessed; a pretrained ViT is adapted via low-rank adapters (LoRA) to minimize trainable parameters; the model is fine-tuned on labeled *Sarcophaga* images, delivering accurate species predictions with high-confidence scores, ensuring robust performance on small datasets.

To evaluate the trade-offs between classification accuracy and computational efficiency, six widely used deep-learning models were selected, including ResNet50, SE-ResNet50, DenseNet121, VGG19, AlexNet, and ViT-LoRA. Convolutional neural networks such as ResNet and DenseNet have demonstrated strong performance across a broad spectrum of visual recognition tasks. However, the inherent inductive biases such as locality and translation invariance restrict their ability to capture long-range dependencies and complex global patterns within images. In contrast, ViT architectures rely entirely on self-attention mechanisms, and have shown remarkable accuracy and generalization in various tasks [30]. Nevertheless, ViT typically requires large-scale training datasets and prolonged training times [33]. In scenarios, the scarcity of data can hinder the effective training and lead to overfitting or training instability. To overcome these limitations, LoRA is a fine-tuning technique that can significantly reduce the number of trainable parameters by introducing low-rank decomposition into selected layers of a pre-trained model [34]. Instead of updating all model weights, LoRA optimizes a compact set of parameters, enabling efficient adaptation to downstream tasks with minimal computational overhead. This makes it particularly suitable for domains characterized by limited data availability and constrained computational resources. LoRA thus facilitates practical deployment of transformer-based models in settings where considerations such as memory usage, inference latency, and GPU availability are critical [35]. In this study, we employed a ViT model pre-trained on the large-scale ImageNet dataset, leveraging its ability to learn generalized visual representations (Figure 1). To adapt it to our task-specific dataset, we applied LoRA during fine-tuning, updating only the low-rank adaptation modules and the classification head. This parameter-efficient strategy enabled rapid convergence, reduced the risk of overfitting, and maintained high classification performance even on a relatively small dataset size, making it an ideal solution for species identification of flesh flies.

Prior to training, we retained the original high-resolution images and applied preprocessing in a split-aware manner. Data augmentation was applied only to the training set. Specifically, for each training image, we performed random geometric and photometric augmentations, followed by a random crop at the original resolution to avoid introducing fixed-size biases. After augmentation, images were resized to the model-specific input resolution; all CNN baselines were trained with 224 × 224 inputs, whereas ViT-LoRA used 384 × 384 inputs to match its architecture. In contrast, the validation/test images were not augmented; they were only resized to the corresponding input size for each model. All models were trained using identical optimization settings. Training was performed for 50 epochs using stochastic gradient descent (SGD) optimization with an initial learning rate of 0.001 and incorporated batch normalization on Nvidia RTX 3060 GPUs (Santa Clara, CA, USA) [36,37]. Performance was evaluated on the held-out test set using precision, recall, accuracy, and F1 score. Additionally, inference speed was also quantified by measuring the average prediction time per image on the test dataset. To further assess classification performance and misclassification patterns, confusion matrices were generated, providing a detailed visualization of per-class accuracy. Ultimately, the best-performing model was subsequently deployed in a web-based application for automated species identification.

## 3. Results

### 3.1. Model Complexity and Performance

Among the conventional CNN-based architectures, DenseNet121 provided a relatively favorable balance between model size and classification performance. On our dataset, it achieved 95.86% accuracy with 6.97 M parameters and a model size of approximately 27.1 MB, suggesting that it may be suitable for deployment in edge or resource-constrained environments. By contrast, VGG19 showed substantially higher model complexity, with 134.33 M parameters and a model size of approximately 512 MB, while offering only limited performance gains relative to its computational cost. AlexNet and ResNet50 also showed less favorable trade-offs between model complexity and classification accuracy. Notably, ViT-LoRA distinguished itself by requiring only approximately 0.16 M trainable parameters, the lowest number among the evaluated deep-learning architectures, while still achieving the best overall classification performance (Table 2 and Table 3). This result highlights the advantage of parameter-efficient fine-tuning, which enables effective transfer learning with substantially reduced training overhead. In addition, the use of an optimized data loader and an object-oriented image-processing pipeline improved the efficiency of data handling and facilitated systematic evaluation of different preprocessing strategies and training hyperparameters, including crop strategy, augmentation settings, input resolution, learning rate, and fine-tuning settings. Together, these results suggest that ViT-LoRA provides a favorable combination of classification accuracy, parameter efficiency, and scalability for species identification of flesh flies.

### 3.2. Confusion Matrix Comparison

**Figure 2:**
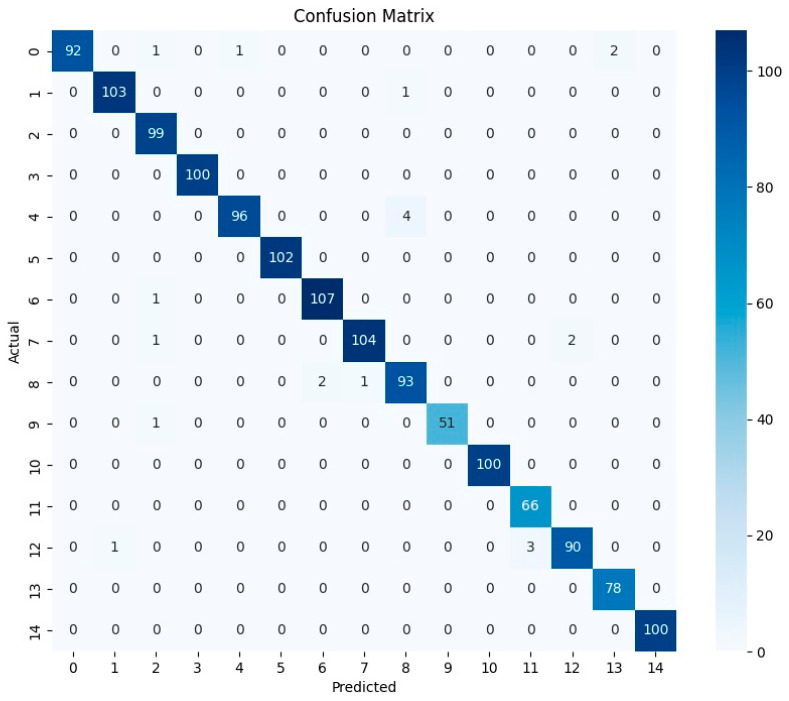
Confusion matrix of the ViT-LoRA model for identifying 15 *Sarcophaga* species. Correct predictions are shown on the main diagonal, with stronger concentrations along this diagonal indicating higher accuracy. Off-diagonal errors occur between *S. dux* and *S. polystylata*, consistent with potential morphological similarity. Each number on the axes represents each species. Note: 0: *S. misera*, 1: *S. albiceps*, 2: *S. similis*, 3: *S. dux*, 4: *S. peregrina*, 5: *S. melanura*, 6: *S. tuberosa*, 7: *S. kanoi*, 8: *S. africa*, 9: *S. crassipalpis*, 10: *S. brevicornis*, 11: *S. polystylata*, 12: *S. pingi*, 13: *S. cinerea*, 14: *S. pterygota*.

Confusion matrices provide a class-by-class summary of model predictions by comparing the true labels with the predicted labels for all test samples (Figure 2 and Figures S3–S7). In these matrices, rows represent the ground-truth classes and columns represent the predicted classes. Correct predictions are concentrated on the main diagonal, whereas off-diagonal entries indicate misclassification between species. Therefore, stronger diagonal dominance reflects better discriminatory performance, particularly in fine-grained classification tasks involving morphologically similar taxa. Among these models, ViT-LoRA exhibited the strongest diagonal dominance, with only slight confusion involving *S. dux* and *S. polystylata*. This result indicates that ViT-LoRA achieved the most accurate and stable species discrimination among the evaluated architectures. Its strong performance likely reflects the ability of the transformer-based framework with low-rank adaptation to capture both global image context and subtle interspecific morphological differences. SE-ResNet50 also showed a strong diagonal pattern, although slight misclassification was observed for *S. albiceps*, *S. melanura*, *S. brevicornis* and *S. polystylata*, indicating comparatively strong class-level performance. VGG19 displayed a moderately diagonal matrix, with more frequent confusion involving *S. misera*, *S. albiceps* and *S. dux*. DenseNet121, ResNet50, and AlexNet exhibited broader off-diagonal distributions, suggesting lower discriminatory power for several closely related *Sarcophaga* species. Overall, the confusion-matrix analysis was consistent with the quantitative evaluation metrics and further supports the superior performance of ViT-LoRA in the fine-grained classification of *Sarcophaga* species.

### 3.3. Training Loss Curve Evaluation

**Figure 3:**
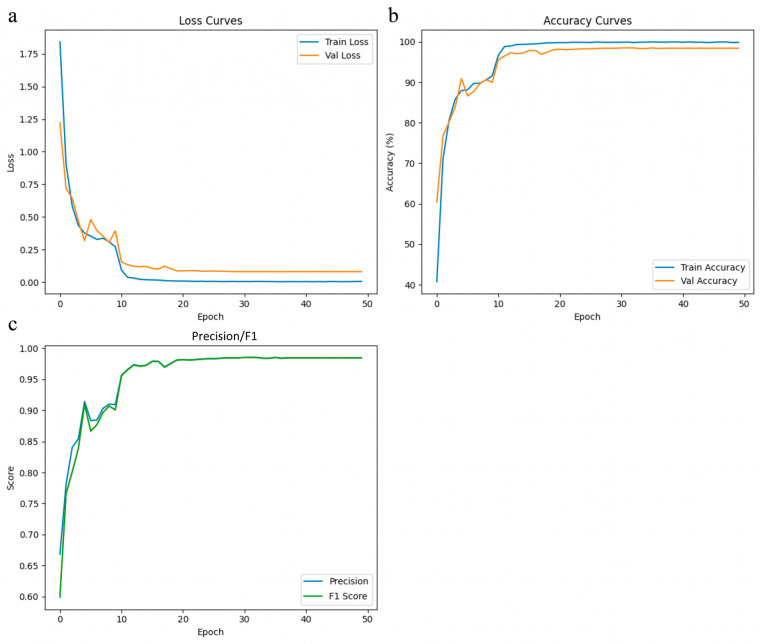
Training dynamics of the ViT-LoRA model for species-level identification of 15 *Sarcophaga* species. (**a**) The training and validation loss curves exhibit a steep early decline, stabilizing with a small generalization gap. (**b**) The training and validation accuracy rise rapidly, plateauing at nearly 99% by approximately 15 epochs. (**c**) Macro-averaged precision, recall, and F1 scores increase in tandem, stabilizing at around 0.98, indicating balanced, robust performance with no overfitting under data-limited conditions.

Training loss curves summarize the optimization process by measuring the discrepancy between predicted outputs and true labels over successive epochs (Figure 3 and Figures S8–S12). A rapidly decreasing and stable loss curve generally indicates efficient fitting to the training data and effective optimization. ViT-LoRA exhibited the steepest loss decline during the early stage of training and reached a stable plateau within approximately 10 epochs, indicating rapid convergence and high learning efficiency. This pattern is consistent with the use of a pretrained transformer backbone combined with parameter-efficient fine-tuning, which enables effective adaptation under limited-data conditions. SE-ResNet50 also showed fast convergence with a smooth and stable loss trajectory throughout training, suggesting robust optimization behavior. ResNet50 displayed a more gradual but still stable decrease in training loss, indicating steady feature learning but a slower convergence rate than the top-performing models. In contrast, VGG19 showed more pronounced fluctuations during training, suggesting greater sensitivity to optimization settings. DenseNet121 converged more slowly than ViT-LoRA and SE-ResNet50, although its loss curve remained generally stable. AlexNet maintained a comparatively higher training loss across epochs, indicating more limited learning capacity for this fine-grained multi-class classification task.

### 3.4. Accuracy Curve Comparison

Accuracy curves provide insight into model learning progression and classification reliability across training epochs (Figure 3 and Figures S8–S12). ViT-LoRA exceeded 90% accuracy within the first 10 epochs and then stabilized at a high level, indicating efficient adaptation and rapid convergence despite having substantially fewer trainable parameters than several CNN-based models. SE-ResNet50 also reached a high accuracy of approximately 97.50% and remained stable throughout the later training epochs, indicating strong classification consistency. DenseNet121 converged to a stable accuracy of around 95.86%, suggesting moderate but reliable classification performance. VGG19 achieved a peak accuracy of approximately 97.43%, but exhibited more noticeable oscillations across epochs, implying less stable optimization despite its strong final performance. By comparison, ResNet50 reached an accuracy of around 93.15%, while AlexNet achieved a maximum accuracy of 93.37%, with both models showing lower overall performance than ViT-LoRA, SE-ResNet50, and VGG19. The larger fluctuations observed in AlexNet further suggest reduced stability in handling this fine-grained multi-class classification task.

### 3.5. Performance Metrics

A comprehensive assessment based on standard classification metrics, including accuracy, precision, recall, and F1 score, showed that ViT-LoRA achieved the best overall performance, with all four metrics reaching 98.5% (Table 3). These results support its strong classification capability under limited-data conditions. SE-ResNet50 followed closely, with all metrics around 97.5%, indicating balanced and stable performance. VGG19 also achieved similarly high values (approximately 97.4%), although its substantially larger parameter budget reduced its practical efficiency relative to the modest performance gain obtained. DenseNet121 reached approximately 95.86% across the evaluation metrics, indicating acceptable but clearly lower fine-grained discrimination than the top two models. AlexNet achieved an accuracy of 93.37%, slightly exceeding that of ResNet50 (93.15%), but both models showed lower overall performance than ViT-LoRA, SE-ResNet50, and VGG19. Taken together, these results indicate that ViT-LoRA offers the strongest balance between predictive performance and parameter efficiency for fine-grained identification of *Sarcophaga* species.

### 3.6. Web-Based Application for Automated Fly Species Identification

Based on the mentioned as above, the best-performing model (ViT-LoRA) was packaged into a web-based application named “FleshFly ID Pro 2.0”, which is accessible via a local URL and enables users to upload adult fly images and obtain the top species prediction with an associated confidence score (Figure S13). However, the present study should primarily be regarded as a methodological study and proof of concept rather than the development of a mature end-user platform. At present, FleshFly ID Pro 2.0 remains a local prototype and has not yet been deployed as a public online system. In addition, the current framework is limited to 15 *Sarcophaga* species, and broader application will require further expansion of taxonomic coverage, specimen number, and image diversity. Despite the promising performance of the selected model, expert taxonomists remain indispensable. Their expertise is essential for specimen identification, dataset curation, result validation, and the continued refinement of automated recognition approaches [22]. Overall, this study provides a preliminary methodological basis for future development of more scalable and standardized image-based identification tools for flesh flies in forensic entomology.

## 4. Discussion

Forensic entomology plays a critical role in the investigation of decomposed corpses, particularly for PMI estimation [8]. Although China encompasses extensive climatic and ecological regions, current studies on the diversity of necrophagous flies have focused largely on inland habitats, whereas coastal regions remain comparatively underexplored [38,39,40,41]. The Shandong Peninsula, bordered by the Bohai Sea to the north and the Yellow Sea to the east, represents a humid warm-temperate monsoon region with distinct coastal environmental conditions. However, faunistic records of necrophagous flies from its coastal zone remain scarce. Therefore, we conducted targeted carrion-baited surveys in coastal areas with dense human activity and recorded 15 *Sarcophaga* species. Notably, *S. cinerea*, *S. pingi*, and *S. pterygota* were newly recorded in this region [32], highlighting the ecological distinctiveness of the area and the significance of entomofaunal surveys in coastal environment. These findings further suggest that climatic differences between coastal and inland areas may significantly affect the species distribution of necrophagous flies. We note, however, that attraction of adults to carrion bait does not necessarily demonstrate larval development on carrion, as some species may visit decomposing tissues primarily for adult feeding or other ecological purposes.

Rapid and accurate species identification is critical for forensic investigations [42]. Traditional morphological diagnosis is hampered by intraspecific variation in body size, color, and shape across developmental stages, as well as the prevalence of cryptic species, all of which hinder reliable identification [15]. Although mitochondrial DNA barcoding and cuticular hydrocarbon profiling provide alternative strategies for identifying forensically important flesh flies, these methods are often destructive, costly, and time-consuming. Wing morphometrics has also been proposed as a supplementary approach; however, species-level classification within the genus *Sarcophaga* remains unreliable due to significant morphological overlap, and closely related species are frequently misclassified even with morphometric features [43]. Recent studies have shown that CNN-based computer vision has considerable potential for species identification in forensic entomology [19,20], but have mainly focused on morphologically distinct taxa. For example, Gohe et al. reported very high classification accuracy for samples from different families, but their task did not address species-level discrimination within a single taxonomically challenging group [27]. Similarly, Ma et al. applied a transformer-based framework to three relatively distinct forensic fly species from Calliphoridae, Sarcophagidae, and Muscidae, rather than to multiple closely related species within the same family [28]. In contrast, our study focused specifically on species-level discrimination within a single morphologically genus, and ViT-LoRA achieved 98.5% accuracy while updating only ~0.16 M trainable parameters, highlighting the effectiveness of parameter-efficient transformer adaptation under limited-data conditions. Nevertheless, the scarcity of high-quality, well-annotated image datasets for forensically important species still limits the rigor and generalizability of image-based identification models. Given these limitations, automated, non-destructive, and scalable methods that can complement human taxonomic expertise remain urgently required.

To further evaluate model performance, we compared ViT-LoRA with five widely used CNN models (ResNet50, SE-ResNet50, DenseNet121, VGG19, AlexNet). While CNNs have been widely applied in biological image classification, which rely heavily on local receptive fields and translation equivariance, and limit their ability to model long-range spatial dependencies. By contrast, ViT-LoRA utilizes global self-attention to integrate scene-level semantics with subtle morphological cues essential for distinguishing closely related fly species [44]. Notably, ViT models typically require large-scale datasets to achieve effective convergence [45]. To overcome this limitation, we therefore evaluated the performance of non-pretrained models and compared them with our parameter-efficient ViT-LoRA fine-tuning. Furthermore, by optimizing the fine-tuning schedule, ViT-LoRA improved pretrained transformer performance and stability [46], underscoring the importance of efficient adaptation strategies in domain-specific, data-limited scenarios. Specifically, LoRA updates only a small set of low-rank adapters within the transformer blocks, which reduces trainable parameters and computational burden while preserving representational capacity. As shown in the confusion matrix, ViT-LoRA achieved strong diagonal dominance, indicating highly accurate predictions across all classes. In contrast, models such as SE-ResNet50, ResNet50, and AlexNet exhibited broader off-diagonal errors, indicating difficulty modeling complex inter-class boundaries. The training loss curve further corroborated these results, with ViT-LoRA demonstrating a steep decline and stabilizing within ~10 epochs, consistent with fast and stable convergence. Notably, ViT-LoRA required only ~0.16 M trainable parameters due to the low-rank adapters, enabling lightweight transfer learning and scalable deployment in data-limited conditions. Despite being trained on a relatively small dataset, ViT-LoRA consistently outperformed all five CNN models in terms of classification accuracy, robustness, and generalization.

To bridge the gap, we integrated ViT–LoRA into a web-based identification platform named “FleshFly ID Pro 2.0”. The system enables users to upload images of adult flesh flies and receive species-level predictions with associated confidence scores. This interactive feedback offers a valuable tool for forensic investigators in order to achieve rapid and accurate species identification. However, the platform remains limited; it is accessible only via desktop browsers and is limited to 15 *Sarcophaga* species. Meanwhile, this prototype should be regarded only as a proof of concept rather than a mature end-user platform. The main purpose of the present study was to establish and evaluate an image-based identification framework and to compare alternative deep-learning architectures, rather than to develop a fully operational deployment system. To enhance scalability and validity, the dataset should be expanded to contain additional species, as well as the specimens from various developmental stages and geographically diverse populations. The model should be further refined through continued fine-tuning and domain adaptation to enhance generalization under variable imaging conditions. A cloud-based API should also be deployed to support remote access and facilitate integration into broader systems. Additionally, we also integrate with mobile applications to enable real-time species identification in field settings. This multi-phase roadmap aligns with emerging trends in biodiversity informatics, mobile diagnostics, and lays the foundation for more accurate, efficient, and reliable identification tools in the future.

## 5. Conclusions

This study represents a targeted coastal survey of *Sarcophaga* species along the Shandong Peninsula, indicating that coastal ecological conditions are associated with geographical shifts in species composition and distribution. To solve the challenge of species identification, we further developed an automated classification framework based on ViT-LoRA, which outperformed the evaluated CNN-based models in both accuracy and efficiency. These findings highlight the potential of ViT-LoRA model for species identification under limited-data conditions. Automated classification should be regarded as a supportive tool rather than a replacement for expert taxonomy.

## Figures and Tables

**Table 1:** Sampling sites for 82 adult specimens representing 15 *Sarcophaga* species, collected along the coastal Shandong Peninsula, China.

No.	Subgenus	Species	ID of Specimen	Collected Location	Date
01	*Parasarcophaga*	*Sarcophaga misera* Walker, 1849	H01 (0)♂	Dishui Wetland Park [119°31′ E, 35°09′ N]	2024.6
			H02 (0)♂	Qingdao Golden Beach Scenic Area [120°24′ E, 35°95′ N]	2025.7
			H03 (0)♂	Shiwan Forest Park [120°51′ E, 36°11′ N]	2024.7
			H04 (0)♀	Olympic Water Sports Park [119°56′ E, 35°42′ N]	2025.6
			H05 (0)♀	Darushan Scenic Area [121°52′ E, 36°78′ N]	2024.8
			H06 (0)♀	Chashan Scenic Area [122°32′ E, 36°85′ N]	2024.8
02	*Parasarcophaga*	*Sarcophaga albiceps* Meigen, 1826	I01 (1)♂	Olympic Water Sports Park [119°56′ E, 35°42′ N]	2025.6
			I02 (1)♂	Laoshan Scenic Area [120°58′ E, 36°21′ N]	2024.7
			I03 (1)♂	Chengtoushan Scenic Area [122°69′ E, 37°41′ N]	2024.8
			I04 (1)♀	Songhubay Park [119°39′ E, 35°21′ N]	2025.6
			I05 (1)♀	Liugong Island [122°19′ E, 37°50′ N]	2025.8
			I06 (1)♀	Haichang Fisherman’s Wharf [122°19′ E, 37°50′ N]	2024.9
03	*Pandelleisca*	*Sarcophaga similis* (Meade, 1876)	J01 (2)♂	Songhubay Park [119°39′ E, 35°21′ N]	2024.6
			J02 (2)♂	Liugong Island [122°19′ E, 37°50′ N]	2025.8
			J03(2)♂	Zhifu Island [121°39′ E, 37°61′ N]	2024.9
			J04 (2)♀	Rizhao Seaside Forest Park [119°63′ E, 35°54′ N]	2024.6
			J05 (2)♀	Darushan Scenic Area [121°52′ E, 36°78′ N]	2025.8
			J 06 (2)♀	Chengtoushan Scenic Area [122°69′ E, 37°41′ N]	2025.8
04	*Liosarcophaga*	*Sarcophaga dux* Thomson, 1869	K01 (3)♂	Songhubay Park [119°39′ E, 35°21′ N]	2024.6
			K02 (3)♂	Chengtoushan Scenic Area [122°69′ E, 37°41′ N]	2025.8
			K03 (3)♂	Yellow River Estuary Ecotourism Area [119°14′ E, 37°73′ N]	2025.10
			K04 (3)♀	Olympic Water Sports Park [119°56′ E, 35°42′ N]	2024.6
			K05 (3)♀	Haichang Fisherman’s Wharf [122°19′ E, 37°50′ N]	2025.9
			K06 (3)♀	Penglai Sanxianshan Scenic Area [120°75′ E, 37°83′ N]	2024.9
05	*Boettcherisca*	*Sarcophaga peregrina* (Robineau-Desvoidy, 1830)	L01 (4)♂	Olympic Water Sports Park [119°56′ E, 35°42′ N]	2024.6
			L02 (4)♂	Rizhao Seaside National Forest Park [119°63′ E, 35°54′ N]	2025.6
			L03 (4)♂	Shiwan Forest Park [120°51′ E, 36°11′ N]	2024.7
			L04 (4)♀	Laoshan Scenic Area [120°58′ E, 36°21′ N]	2025.7
			L05 (4)♀	Haichang Fisherman’s Wharf [122°19′ E, 37°50′ N]	2024.9
			L06 (4)♀	Yellow River Estuary Ecotourism Area [119°14′ E, 37°73′ N]	2024.10
06	*Helicophagella*	*Sarcophaga melanura* Meigen, 1826	M01 (5)♂	Rizhao Seaside National Forest Park [119°63′ E, 35°54′ N]	2024.6
			M02 (5)♂	Qingdao Golden Beach Scenic Area [120°24′ E, 35°95′ N]	2025.7
			M03 (5)♂	Haichang Fisherman’s Wharf [122°19′ E, 37°50′ N]	2024.9
			M04 (5)♀	Yellow River Estuary Ecotourism Area [119°14′ E, 37°73′ N]	2025.10
			M05 (5)♀	Rizhao Seaside National Forest Park [119°63′ E, 35°54′ N]	2024.6
			M06 (5)♀	Penglai Sanxianshan Scenic Area [120°75′ E, 37°83′ N]	2025.9
07	*Liosarcophaga*	*Sarcophaga tuberosa* Pandelle, 1896	N01 (6)♂	Dishui Wetland Park [119°31′ E, 35°09′ N]	2024.6
			N02 (6)♂	Laoshan Scenic Area [120°58′ E, 36°21′ N]	2024.7
			N03 (6)♂	Shouguang Port [118°99′ E, 37°27′ N]	2025.10
			N04 (6)♀	Songhubay Park [119°39′ E, 35°21′ N]	2024.6
			N05 (6)♀	Darushan Scenic Area [121°52′ E, 36°78′ N]	2024.8
			N06 (6)♀	Liugong Island [122°19′ E, 37°50′ N]	2024.8
08	*Kanoisca*	*Sarcophaga kanoi* Park, 1962	O01 (7)♂	Qingdao Golden Beach Scenic Area [120°24′ E, 35°95′ N]	2024.7
			O02 (7)♂	Yellow River Estuary Ecotourism Area [119°14′ E, 37°73′ N]	2024.10
			O03 (7)♂	Penglai Sanxianshan Scenic Area [120°75′ E, 37°83′ N]	2025.9
			O04 (7)♀	Dishui Wetland Park [119°31′ E, 35°09′ N]	2024.6
			O05 (7)♀	Liugong Island [122°19′ E, 37°50′ N]	2024.8
			O06 (7)♀	Yellow River Estuary Ecotourism Area [119°14′ E, 37°73′ N]	2024.10
09	*Bercaea*	*Sarcophaga africa* (Wiedemann, 1824)	P01 (8)♂	Olympic Water Sports Park [119°56′ E, 35°42′ N]	2025.6
			P02 (8)♂	Rizhao Seaside National Forest Park [119°63′ E, 35°54′ N]	2024.6
			P03 (8)♂	Liugong Island [122°19′ E, 37°50′ N]	2025.8
			P04 (8)♀	Rizhao Seaside National Forest Park [119°63′ E, 35°54′ N]	2024.6
			P05 (8)♀	Liugong Island [122°19′ E, 37°50′ N]	2024.8
			P06 (8)♀	Penglai Sanxianshan Scenic Area [120°75′ E, 37°83′ N]	2025.9
10	*Liopygia*	*Sarcophaga crassipalpis* Macquart, 1839	Q01 (9)♂	Chashan Scenic Area [122°32′ E, 36°85′ N]	2024.8
			Q02 (9)♂	Chashan Scenic Area [122°32′ E, 36°85′ N]	2024.8
11	*Liosarcophaga*	*Sarcophaga brevicornis* Ho, 1934	R01 (10)♂	Songhubay Park [119°39′ E, 35°21′ N]	2025.6
			R02 (10)♂	Darushan Scenic Area [121°52′ E, 36°78′ N]	2024.8
			R03 (10)♂	Shouguang Port [118°99′ E, 37°27′ N]	2024.10
			R04 (10)♀	Qingdao Golden Beach Scenic Area [120°24′ E, 35°95′ N]	2025.7
			R05 (10)♀	Chengtoushan Scenic Area [122°69′ E, 37°41′ N]	2024.8
			R06 (10)♀	Penglai Sanxianshan Scenic Area [120°75′ E, 37°83′ N]	2024.9
12	*Pandelleisca*	*Sarcophaga polystylata* Ho, 1934	S01 (11)♂	Laoshan Scenic Area [120°58′ E, 36°21′ N]	2025.7
			S02 (11)♂	Shiwan Forest Park [120°51′ E, 36°11′ N]	2024.7
13	*Parasarcophaga*	*Sarcophaga pingi* Ho, 1934	T01 (12)♂	Shiwan Forest Park [120°51′ E, 36°11′ N]	2024.7
			T02 (12)♂	Haichang Fisherman’s Wharf [122°19′ E, 37°50′ N]	2025.9
			T03 (12)♂	Penglai Sanxianshan Scenic Area [120°75′ E, 37°83′ N]	2024.9
			T04 (12)♀	Olympic Water Sports Park [119°56′ E, 35°42′ N]	2024.6
			T05 (12)♀	Liugong Island [122°19′ E, 37°50′ N]	2025.8
			T06 (12)♀	Haichang Fisherman’s Wharf [122°19′ E, 37°50′ N]	2024.9
14	*Leucomyia*	*Sarcophaga cinerea* (Fabricius, 1794)	U01 (13)♂	Dishui Wetland Park [119°31′ E, 35°09′ N]	2024.6
			U02 (13)♂	Laoshan Scenic Area [120°58′ E, 36°21′ N]	2025.7
			U03 (13)♂	Zhifu Island [121°39′ E, 37°61′ N]	2024.9
			U04 (13)♀	Olympic Water Sports Park [119°56′ E, 35°42′ N]	2024.6
			U05 (13)♀	Darushan Scenic Area [121°52′ E, 36°78′ N]	2025.8
			U06 (13)♀	Liugong Island [122°19′ E, 37°50′ N]	2024.8
15	*Pierretia*	*Sarcophaga pterygota* Thomas, 1949	V01 (14)♂	Dishui Wetland Park [119°31′ E, 35°09′ N]	2024.6
			V02 (14)♂	Chashan Scenic Area [122°32′ E, 36°85′ N]	2024.8
			V03 (14)♂	Haichang Fisherman’s Wharf [122°19′ E, 37°50′ N]	2024.9
			V04 (14)♀	Songhubay Park [119°39′ E, 35°21′ N]	2025.6
			V05 (14)♀	Chengtoushan Scenic Area [122°69′ E, 37°41′ N]	2024.8
			V06 (14)♀	Shouguang Port [118°99′ E, 37°27′ N]	2024.10

Note: In each specimen ID, the uppercase letter represents the species code assigned during model training, and the number in parentheses represents the species index used in the confusion matrix during data analysis.

**Table 2:** Comparison of model complexity and performance across ViT-LoRA and five CNN baselines.

Model	Total Parameters (M)	Trainable Parameters (M)	Model Speed (s)	Model Size (MB)
ViT-LoRA	86.25	0.16	0.042	331
AlexNet	57.07	57.07	0.012	217
SE-ResNet50	28.07	28.07	0.043	107
ResNet50	21.29	21.29	0.027	81.3
DenseNet121	6.97	6.97	0.065	27.1
VGG19	134.33	134.33	0.017	512

Note: Model speed (s) indicates the average inference time per image on the test set.

**Table 3:** Performance comparison of ViT-LoRA and five CNNs on species-level identification of 15 *Sarcophaga* species.

Model	Accuracy	Precision	Recall	F1 Score
ViT-LoRA	0.9850	0.9853	0.9850	0.9850
SE-ResNet50	0.9750	0.9756	0.9750	0.9751
VGG19	0.9743	0.9747	0.9743	0.9744
DenseNet121	0.9586	0.9597	0.9586	0.9588
AlexNet	0.9337	0.9345	0.9337	0.9345
ResNet50	0.9315	0.93316	0.9315	0.9314

## Data Availability

The original contributions presented in this study are included in the article/Supplementary Material. Further inquiries can be directed to the corresponding authors.

## Supplementary Materials

The following supporting information can be downloaded at https://www.mdpi.com/article/10.3390/insects17040374/s1. Figure S1: Coastal records of *Sarcophaga* species on the Jiaodong Peninsula (Shandong, China). Colored symbols indicate the occurrence records of representative *Sarcophaga* species collected in relatively higher numbers in this region during carrion-baited surveys along the Bohai and Yellow Seas. Note: Multiple species may co-occur at the same sampling locality. Figure S2: Representative thumbnails from the curated image set of adult *Sarcophaga* used to train and evaluate the ViT–LRA and five CNNs. Figure S3: Confusion matrix of Alexnet model for identifying 15 *Sarcophaga* species. Figure S4: Confusion matrix of Densenet121 model for identifying 15 *Sarcophaga* species. Figure S5: Confusion matrix of Resnet 50 model for identifying 15 *Sarcophaga* species. Figure S6: Confusion matrix of Se-resnet50 model for identifying 15 *Sarcophaga* species. Figure S7: Confusion matrix of VGG 19 model for identifying 15 *Sarcophaga* species. Figure S8: Training dynamics of the Alexnet model for identifying 15 *Sarcophaga* species. Figure S9: Training dynamics of the Densenet121 model for identifying 15 *Sarcophaga* species. Figure S10: Training dynamics of the Resnet 50 model for identifying 15 *Sarcophaga* species. Figure S11: Training dynamics of the Se-resnet50 model for identifying 15 *Sarcophaga* species. Figure S12: Training dynamics of the VGG 19 model for identifying 15 *Sarcophaga* species. Figure S13: A desktop, locally deployable web-based application powered by ViT-LoRA (“FleshFly ID Pro 2.0”) for automated species-level identification of forensic *Sarcophaga* species. Users upload images of adult flies to receive species predictions along with an associated confidence score. The heatmap visualizes the confidence distribution across species.
