# Supplementary material for: Deep Learning Approach for Species Identification of Forensically Important Sarcophagid flies (Diptera: Sarcophagidae) in China
Source: Insects. 2026 Apr 1;17(4):374. doi: 10.3390/insects17040374 (PMC13116862; doi:10.3390/insects17040374)
Supplement: Supplementary file 1 [file insects-17-00374-s001.zip › insects-4196454-supplementary.pdf]

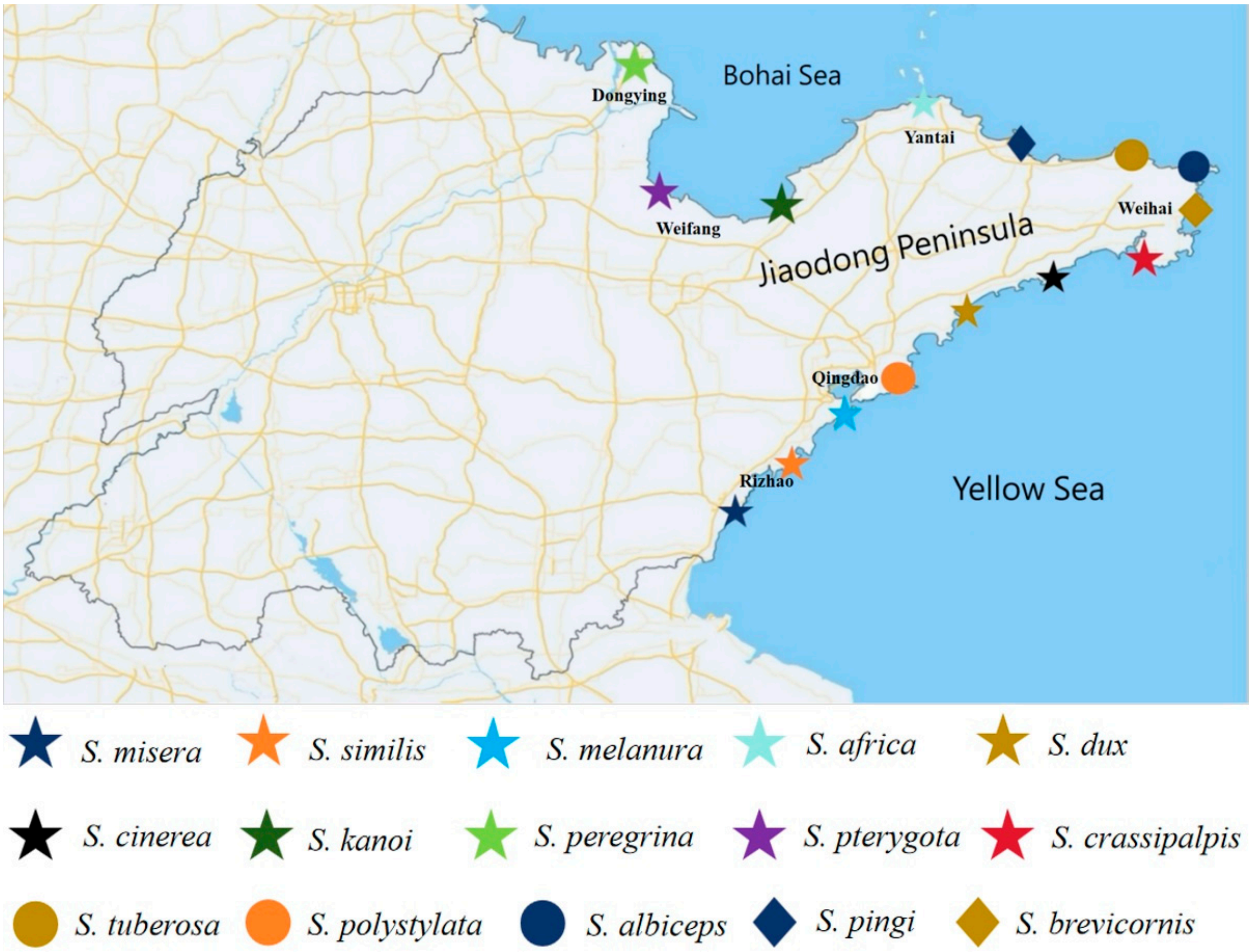

Figure S1 Coastal records of *Sarcophaga* species on the Jiaodong Peninsula (Shandong, China). Colored symbols indicate the occurrence records of representative *Sarcophaga* species collected in relatively higher numbers in this region during carrion-baited surveys along the Bohai and Yellow Seas. Note: Multiple species may co-occur at the same sampling locality.

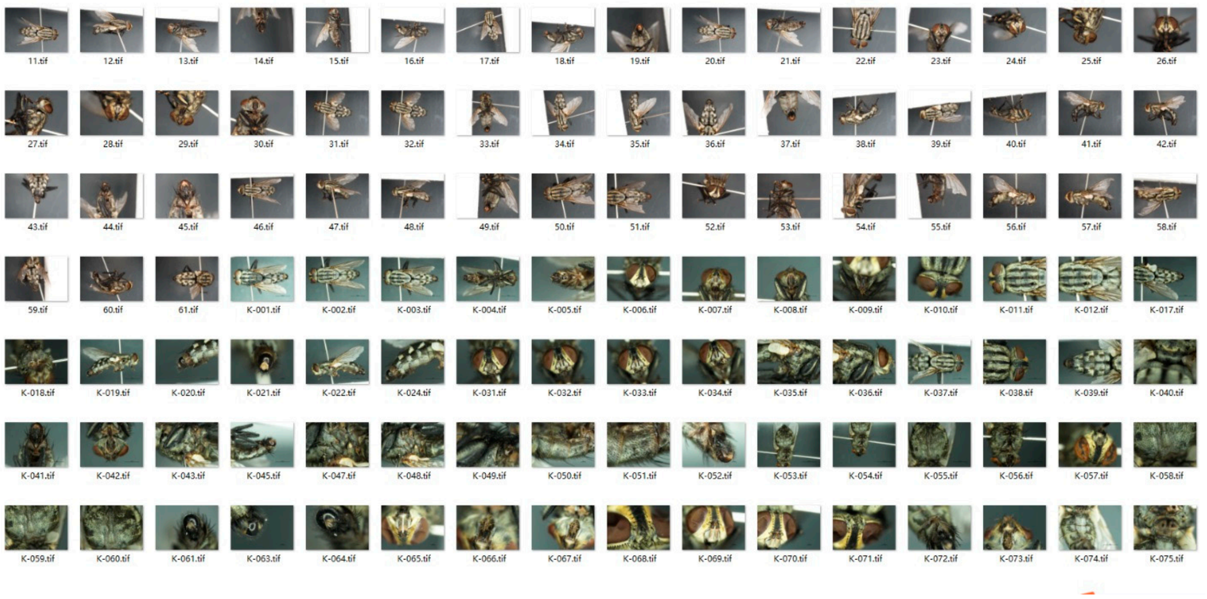

Figure S2 Representative thumbnails from the curated image set of adult *Sarcophaga* used to train and evaluate the ViT–LORA and five CNNs.

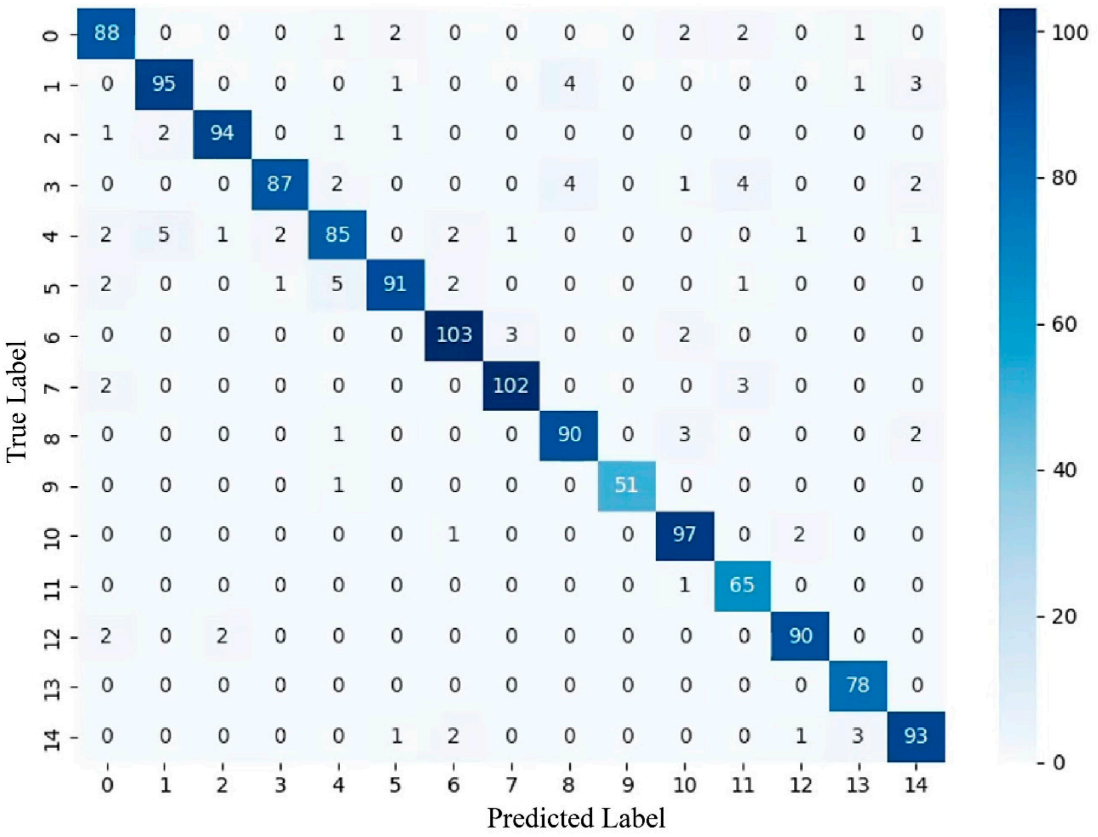

Figure S3 Confusion matrix of Alexnet model for identifying 15 *Sarcophaga* species.

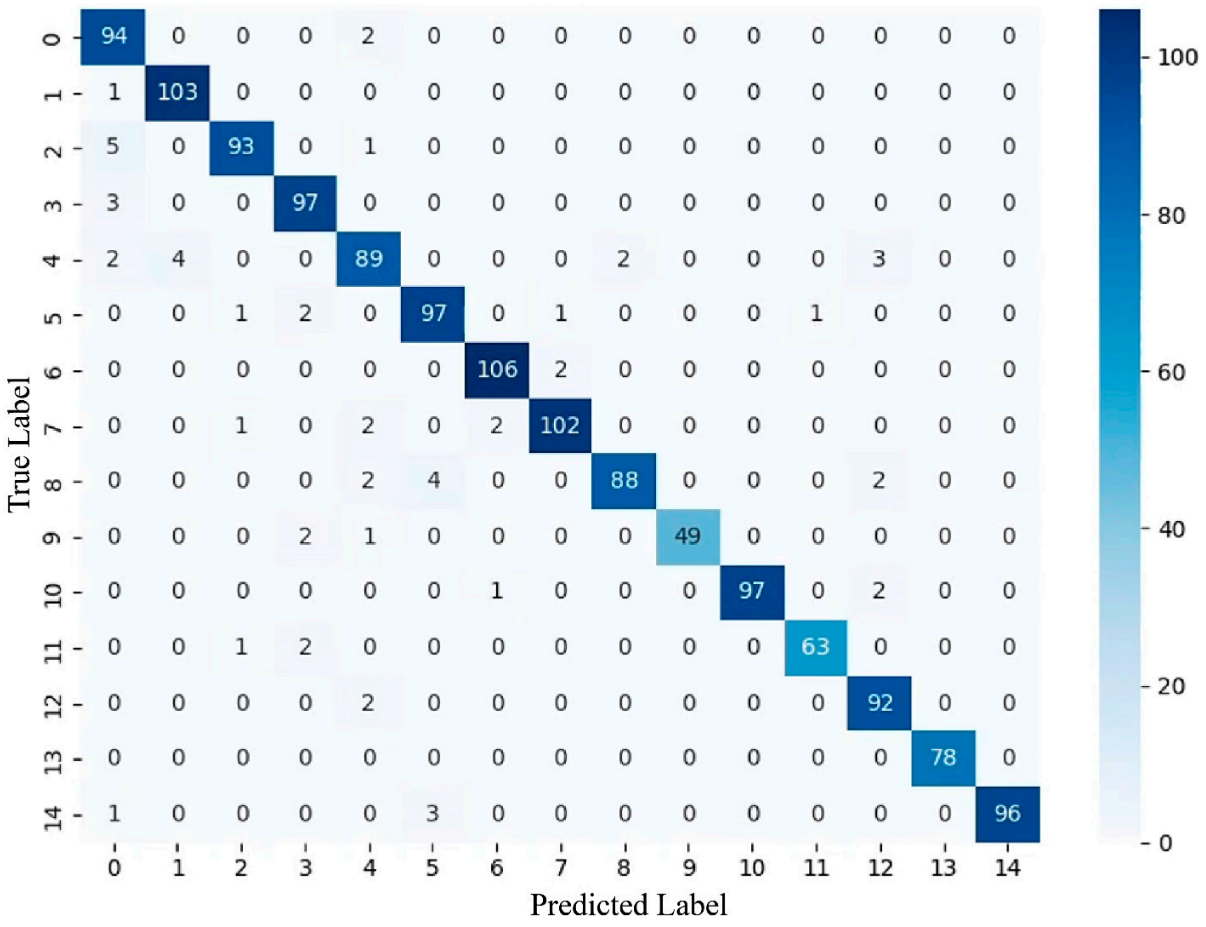

Figure S4 Confusion matrix of Densenet121 model for identifying 15 *Sarcophaga* species.

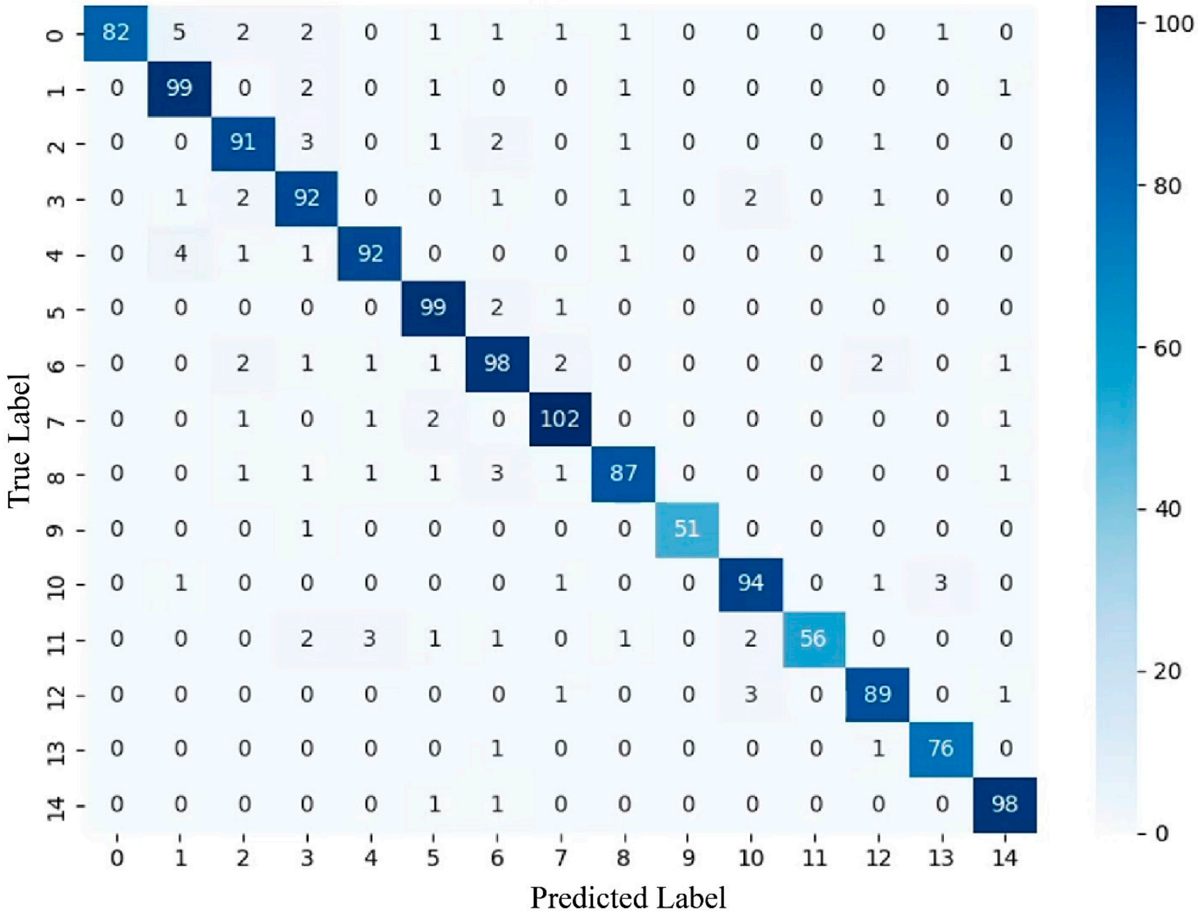

Figure S5 Confusion matrix of Resnet 50 model for identifying 15 *Sarcophaga* species.

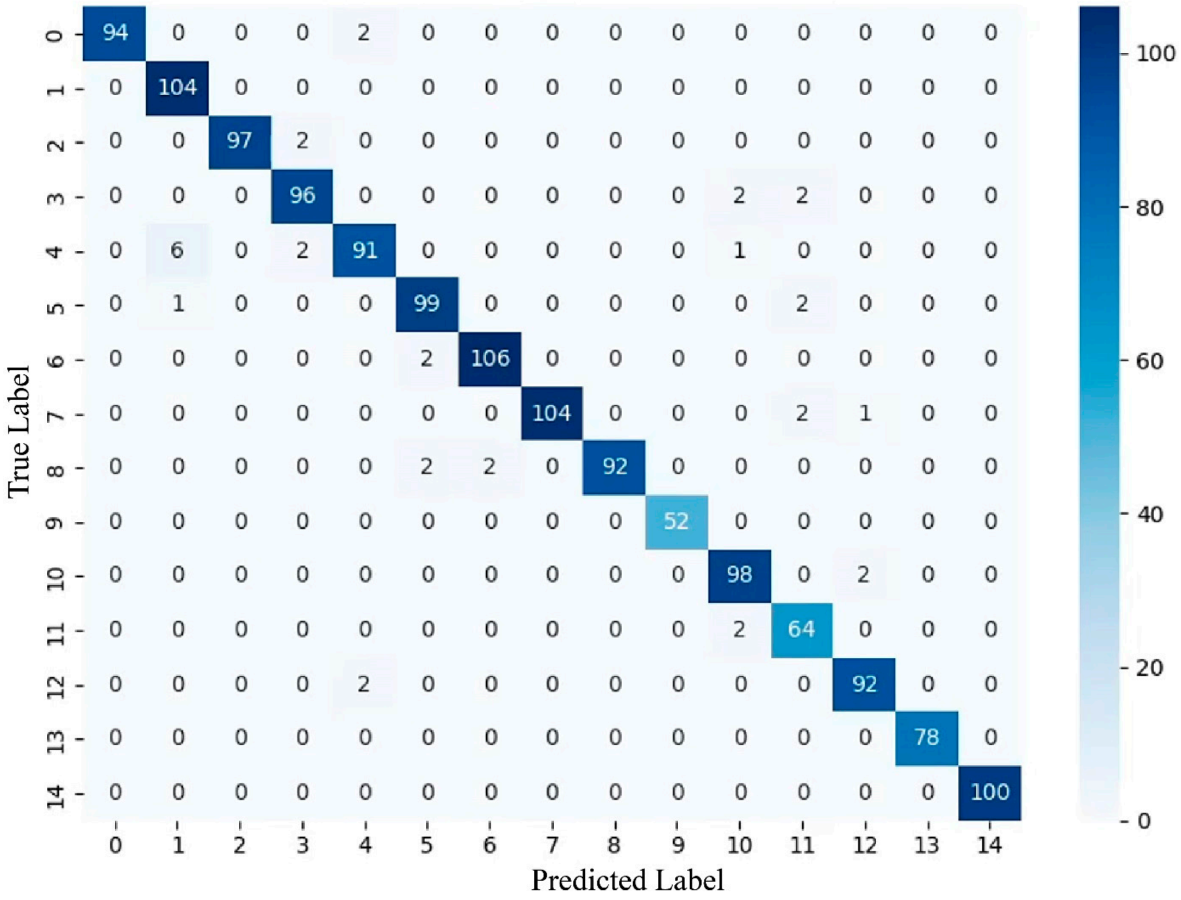

Figure S6 Confusion matrix of Se-resnet50 model for identifying 15 *Sarcophaga* species.

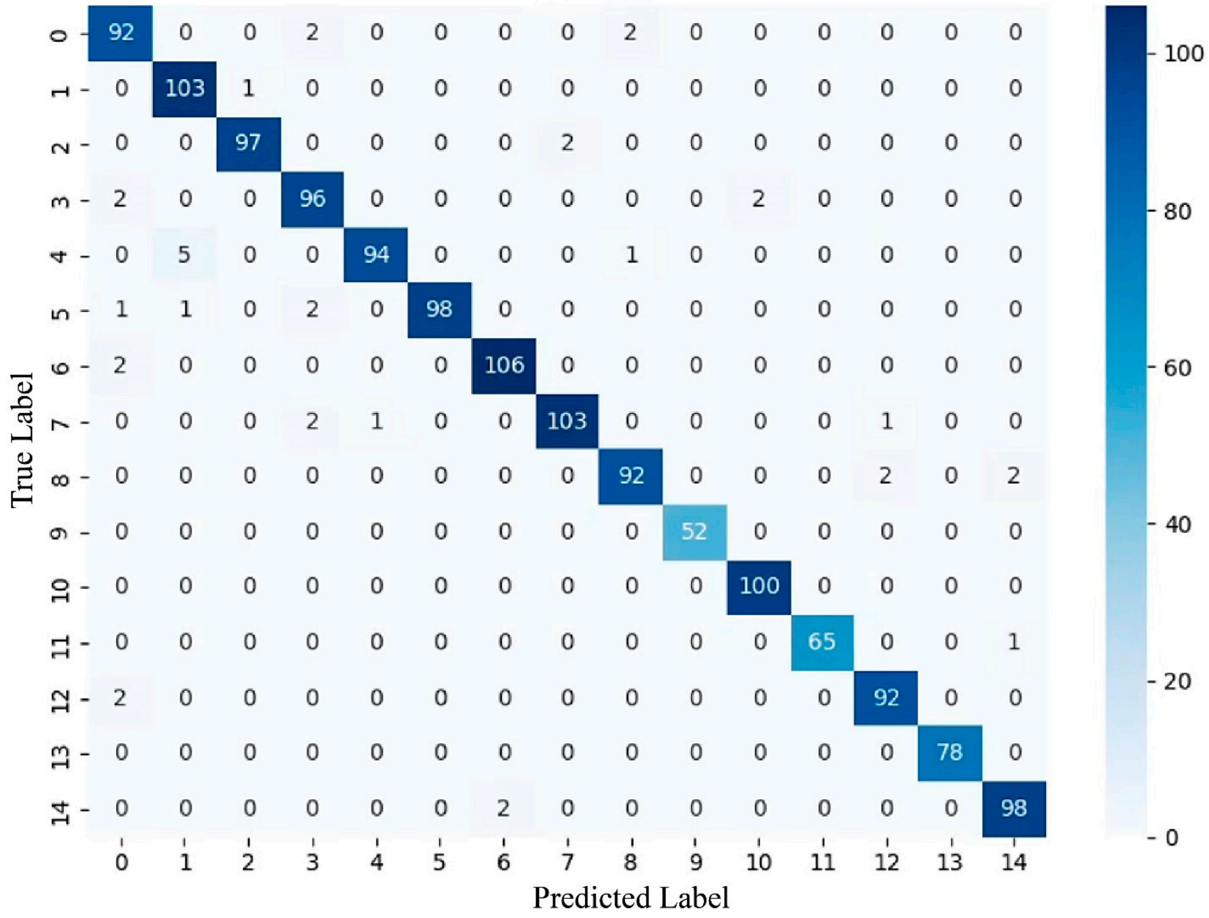

Figure S7 Confusion matrix of VGG 19 model for identifying 15 *Sarcophaga* species.

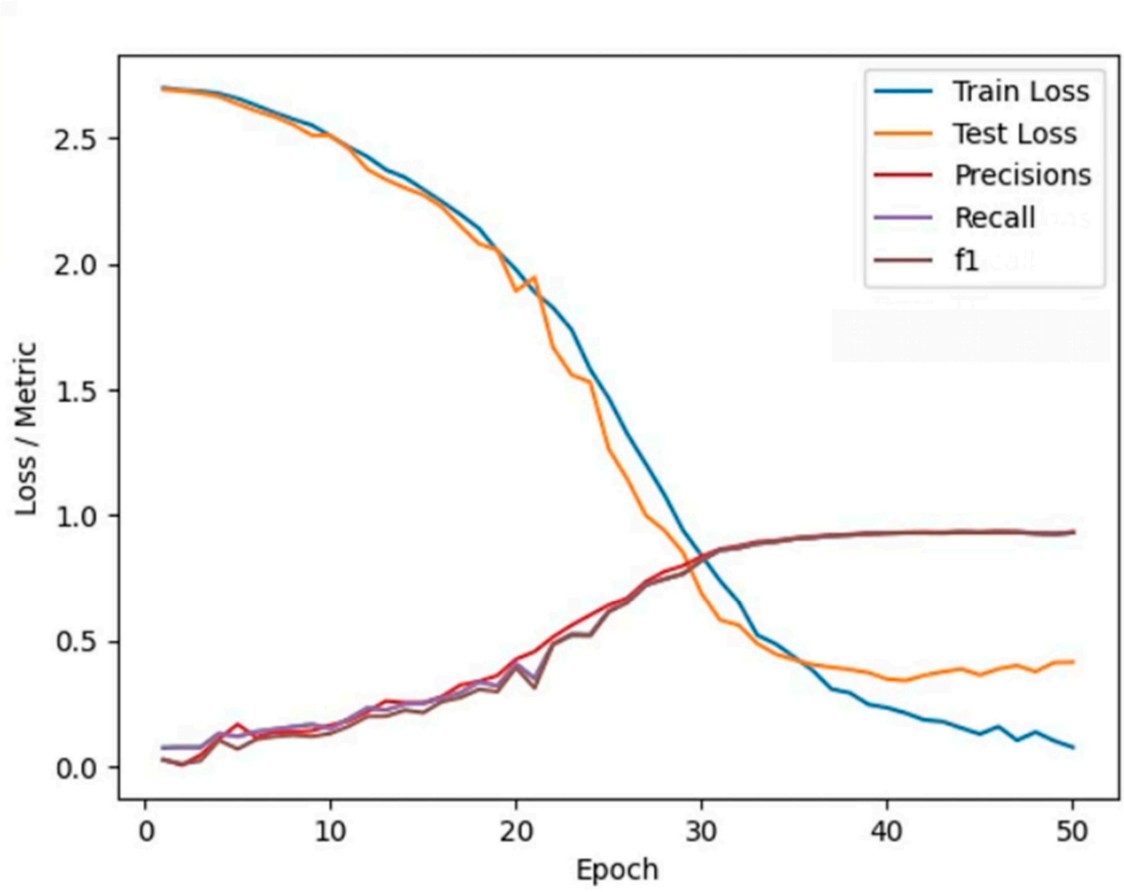

Figure S8 Training dynamics of the Alexnet model for identifying 15 *Sarcophaga* species.

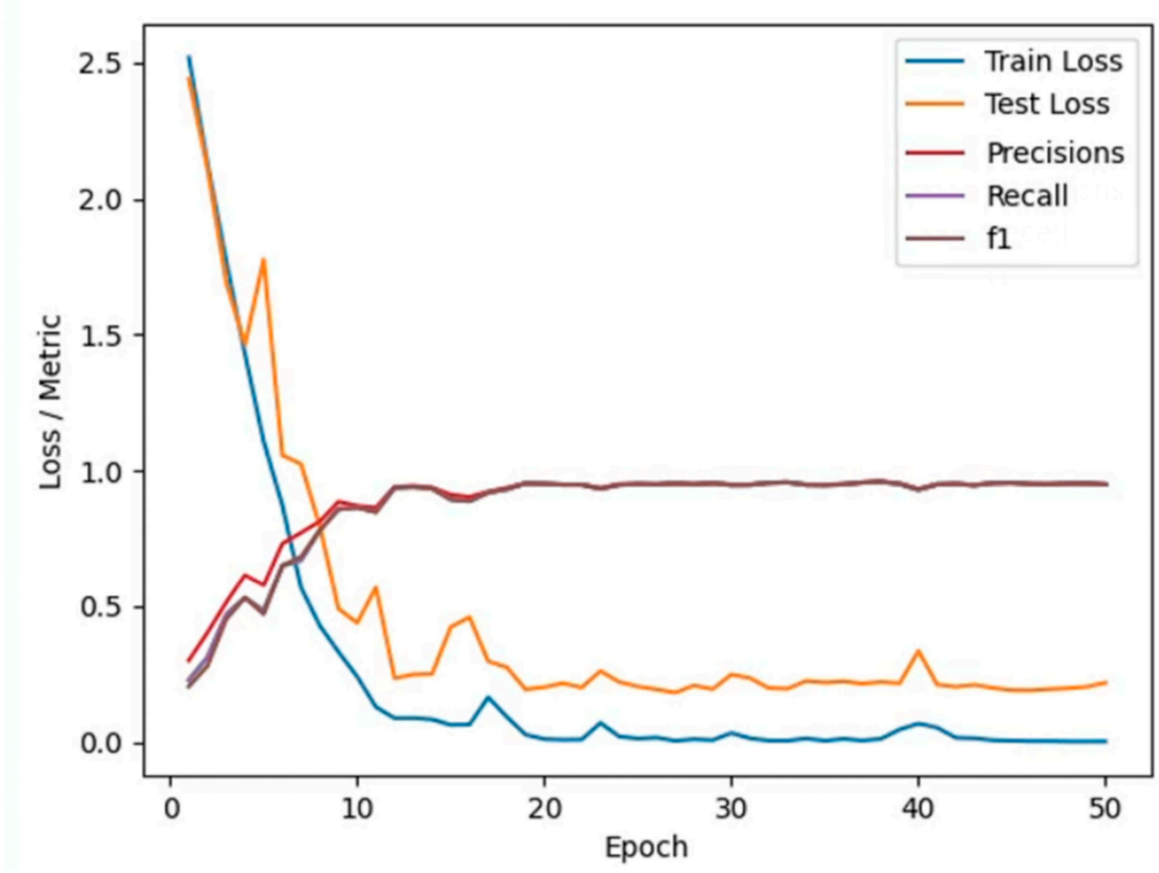

Figure S9 Training dynamics of the Densenet121 model for identifying 15 *Sarcophaga* species.

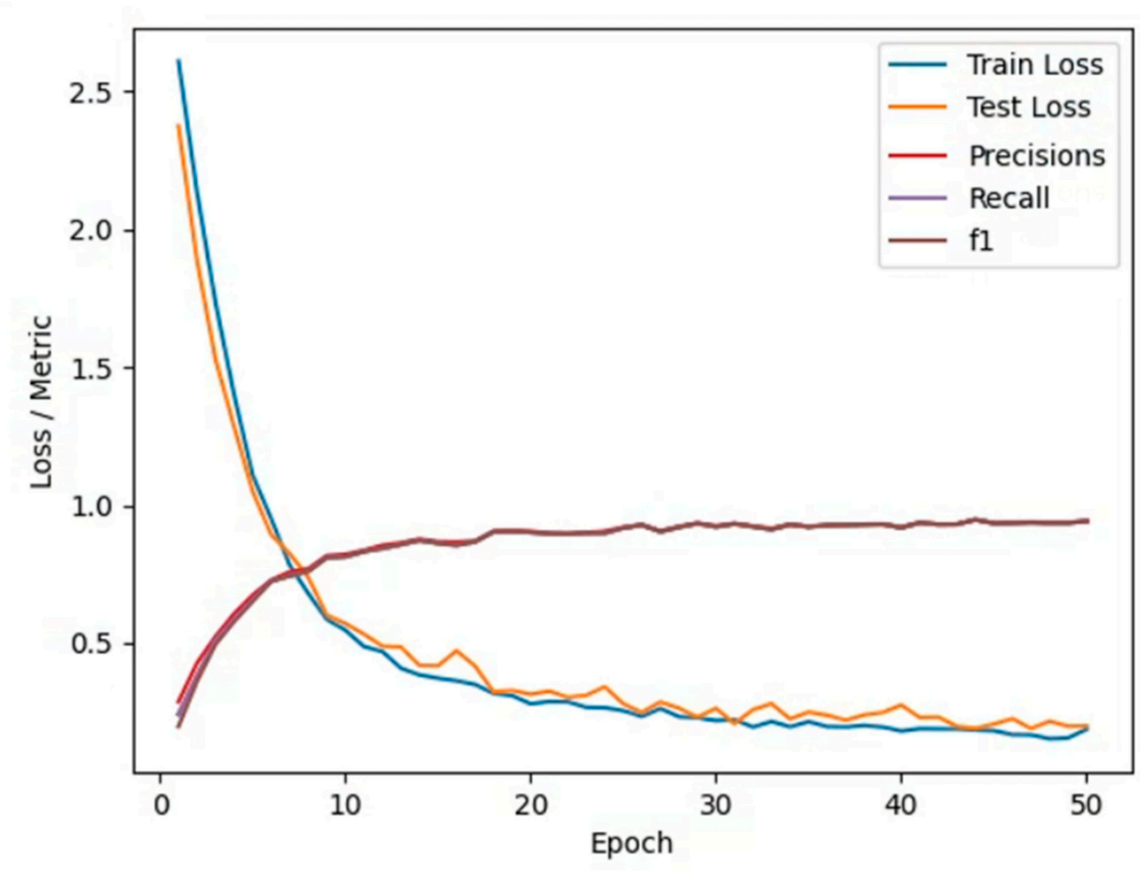

Figure S10 Training dynamics of the Resnet 50 model for identifying 15 *Sarcophaga* species.

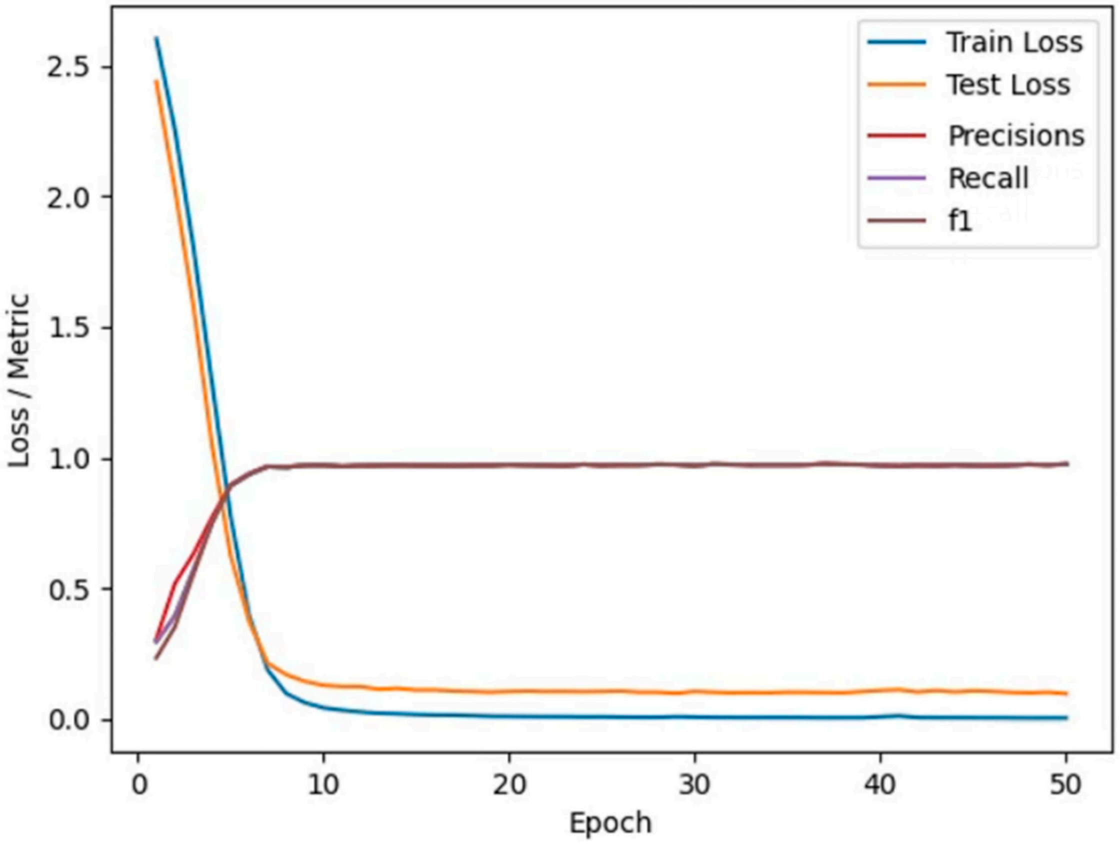

Figure S11 Training dynamics of the Se-resnet50 model for identifying 15 *Sarcophaga* species.

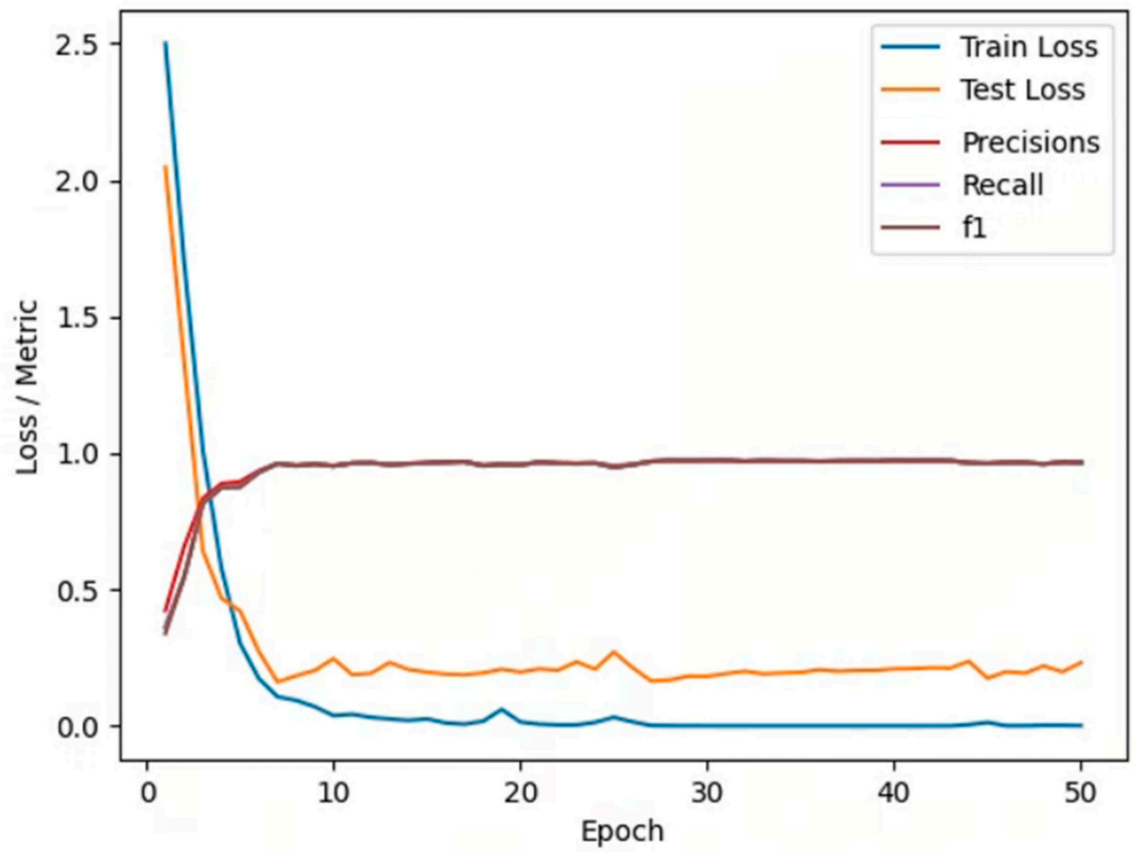

Figure S12 Training dynamics of the VGG 19 model for identifying 15 *Sarcophaga* species.

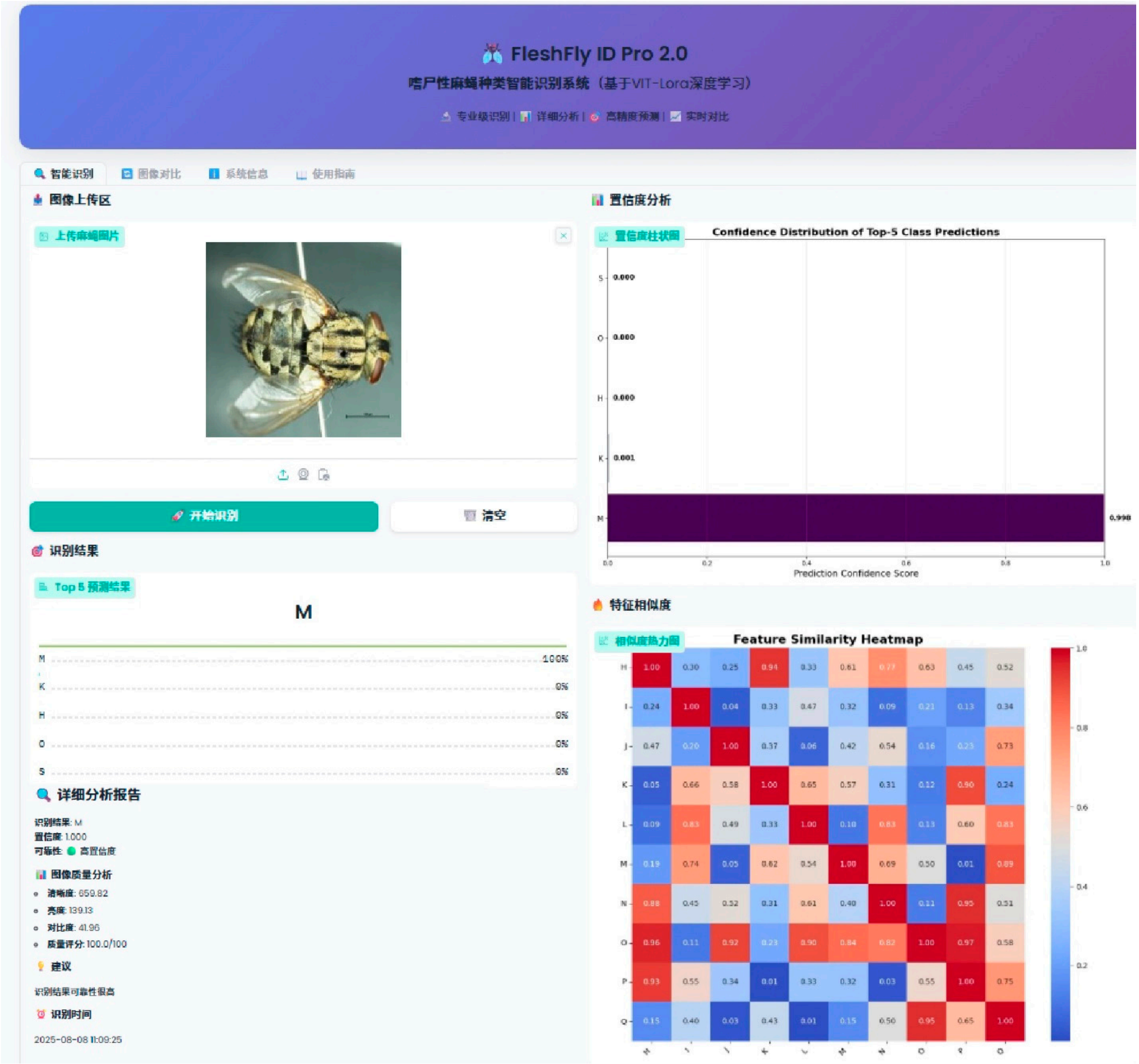

Figure S13 A desktop, locally deployable web-based application powered by ViT-LoRA ("FleshFly ID Pro 2.0") for automated species-level identification of forensic *Sarcophaga* species. Users upload images of adult flies to receive species predictions along with an associated confidence score. The heatmap visualizes the confidence distribution across species.
